# Decoding STAT3: a new frontier in understanding and treating hyperoxic lung injury

**DOI:** 10.3389/fimmu.2025.1657823

**Published:** 2025-09-03

**Authors:** Wulan Li, Qian Liu, Wenyan Xiong, Xu Zhong, Lei Tian

**Affiliations:** ^1^ Department of Anesthesiology, Zigong First People’s Hospital, Zigong Academy of Medical Sciences, Zigong, China; ^2^ Department of Anesthesiology, Yibin Maternity and Children Hospital, Yibin, China; ^3^ The First School of Clinical Medicine, Lanzhou University, Lanzhou, China

**Keywords:** hyperoxia, lung injury, STAT3, inflammation, apoptosis, oxidative stress, therapy

## Abstract

High-concentration oxygen (hyperoxia) therapy is critical for reducing mortality in hypoxemic emergencies, but it can also cause acute and chronic hyperoxic lung injury (HLI), such as diffuse alveolar damage, vascular endothelial injury, and bronchopulmonary dysplasia. Improving the safety of hyperoxia therapy has emerged as an urgent problem. The molecular mechanisms of HLI are not fully understood. Mono-therapy with antioxidant or anti-inflammatory agents has shown limited efficacy in mitigating lethal HLI, highlighting the need for multifaceted treatments. Signal transducer and activator of transcription 3 (STAT3) is involved in anti-inflammatory, anti-apoptotic, and antioxidant processes. Therefore, STAT3-targeted therapy may provide potential benefit in HLI treatment. Substantial evidence indicates that STAT3 is activated in lung cells following hyperoxia exposure and exerts both detrimental and protective effects. Given the increasing insights into STAT3’s role in HLI, a better understanding of the underlying mechanisms is necessary. This review explores the role of the STAT3 pathway in HLI across various cell types and disease models, and highlights recent developments in therapies targeting STAT3. We hope this summary can provide both advancements in understanding the STAT3 signaling pathway and evidence to support the development of novel therapeutic strategies targeting HLI.

## Graphical Abstract

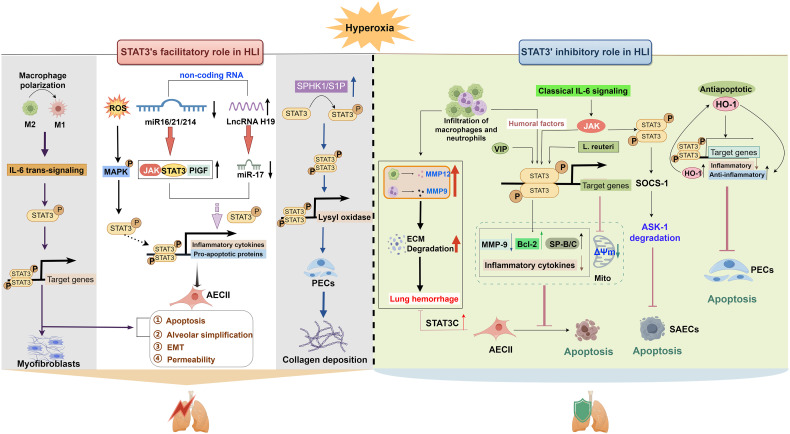

## 1 Introduction

High-concentration oxygen (hyperoxia, FiO2 ≥ 50%) is one of the few available life-saving treatments for critically ill patients with hypoxemia-related respiratory failure, particularly those with acute respiratory distress syndrome (ARDS) and premature infants with immature lungs (1, 2). However, higher oxygen (O_2_) concentrations and prolonged exposure can lead to adverse clinical outcomes, including lung injury, larger myocardial infarcts, retinopathy, and increased morbidity and mortality (3, 4). The lungs are the most seriously damaged organ due to their direct exposure to O_2_. Hyperoxic acute lung injury (HALI), characterized by damage to alveolar epithelial cells (AECs) and pulmonary endothelial cells (PECs), is a leading cause of death from hyperoxia (5). Hyperoxia can also lead to chronic lung injuries like pulmonary fibrosis and bronchopulmonary dysplasia (BPD), characterized by abnormal lung and vascular development (6–8). Despite extensive research, the incidence of HLI remains high due to unclear pathogenesis and lack of effective therapies.

As a widely studied member of the STAT family, signal transducer and activator of transcription 3 (STAT3) is ubiquitously expressed in most tissues. STAT3 is widely recognized for its role in promoting cancer progression and poor prognosis by regulating genes governing tumor survival, growth, invasion, angiogenesis, and drug resistance (9). In pulmonary systems, STAT3 plays a critical role in regulating pathophysiological processes. It influences fundamental cellular processes such as proliferation, differentiation, and apoptosis, along with inflammatory and immune responses. Abnormal activation or suppression of STAT3 is closely linked to the pathogenesis and progression of several pulmonary disorders, such as pulmonary fibrosis, pulmonary infections, and chronic obstructive pulmonary disease (10–12). Oxidative stress, impaired antioxidant defense, inflammation, and programmed cell death constitute core pathological mechanisms in HLI (13, 14). However, the theraputic efficacy of mono-therapy with an antioxidant or anti-inflammatory agent remains limited, reflecting the multifactorial pathogenesis of HLI (15). Given STAT3’s central role in regulating oxidative stress, inflammation, and apoptosis—key drivers of HLI—targeting STAT3 may represent a promising multi-target strategy for HLI therapy.

Recent studies demonstrated that STAT3 is activated in hyperoxia-induced acute and chronic lung injury (16, 17). Targeting STAT3 or its signaling pathway by genetic or pharmacological means has shown a promising therapeutic approach for HLI (18, 19). Given accumulating insights into STAT3’s multifaceted roles in HLI, it is necessary to track and summarize the advancements in this field. This review aims to deepen the understanding of HLI pathogenesis and establish a foundation for developing new preventive and therapeutic options for patients with limited treatment options.

## 2 STAT3 structure, function, and signaling pathways

**Figure 1:**
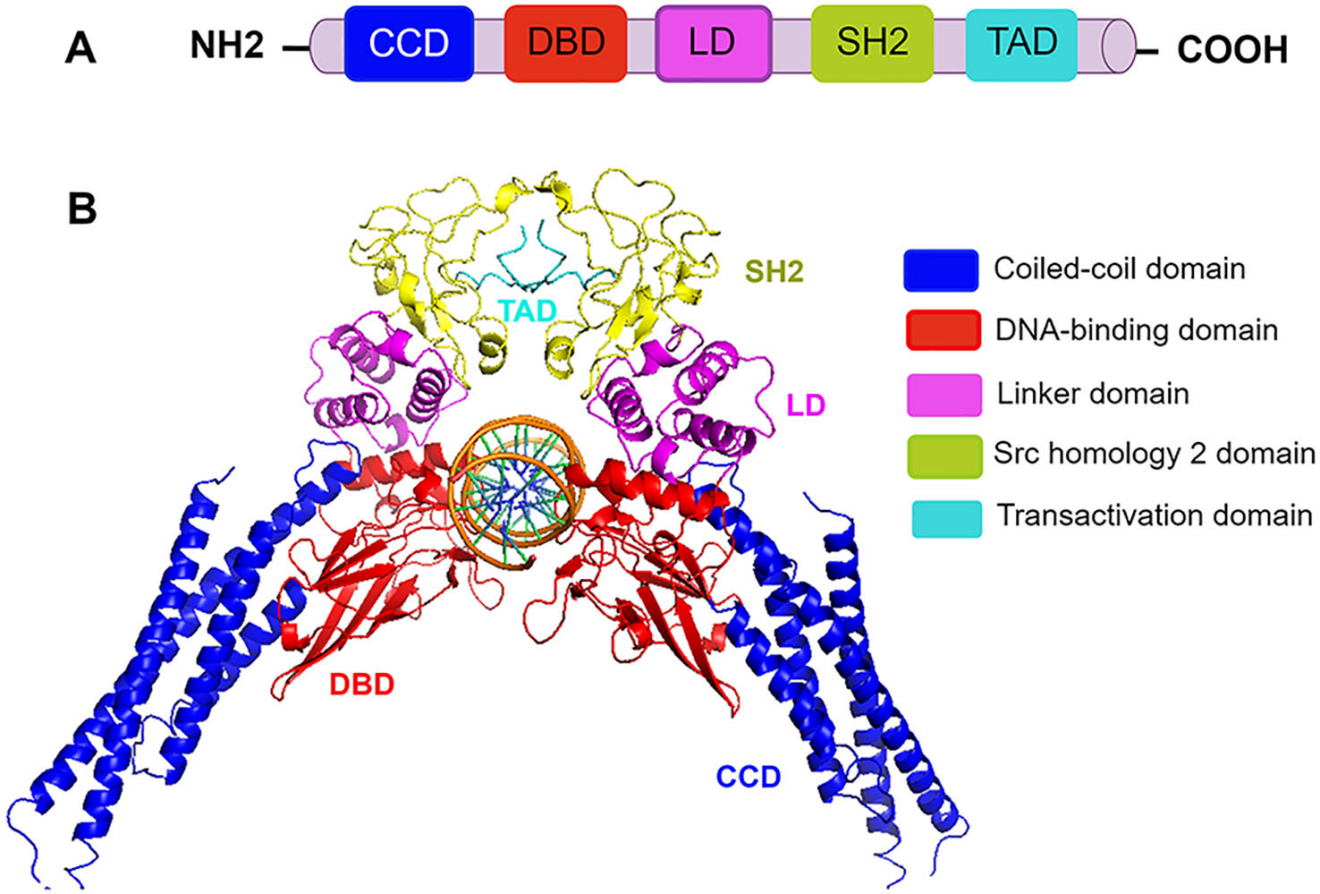
The domain structure of STAT3. **(A)** Schematic domain structure of STAT3. CCD (coiled-coil domain) recruits STAT3 and regulates its phosphorylation, dimerization, and nuclear translocation. The DBD (DNA-binding domain) binds specific DNA sequences. TAD (transactivation domain) drives transcriptional activation. SH2 (Src homology 2) domain mediates STAT3 recruitment, activation, and dimerization through phosphotyrosine residues in the opposing subunit, and a carboxyl-terminal TAD. LD (linker domain) connects DBD to SH2 and ensures the structural stability of the DBD. **(B)** Three-dimensional structure of the STAT3 homodimer bound to DNA (PDB code 1BG1). Structural domains are illustrated by different colors.

STAT3 protein consists of 770 amino acids with six functionally conserved domains. Its core fragment includes the coiled-coil domain (CCD), the DNA-binding domain (DBD), the linker domain (LD), and the Src homology 2 (SH2) domain (12) (**Figure 1**). The CCD, which is composed of four α-helices connected by short loops, primarily recruits STAT3 to its receptor through its large hydrophilic surface (9). The DBD contains an eight-stranded β-barrel and binds specific DNA sequences. The LD, composed of multiple α-helices, bridges the DBD and SH2 domain to maintain DBD structural integrity. The SH2 domain, which is the most conserved region of this family, is essential for its activation and dimerization. It binds tyrosine-phosphorylated residues on cell surface receptors to initiate phosphorylation and subsequently interacts with phosphorylated tyrosine (Tyr705) on another STAT3 molecule to form active dimers. Inhibiting the SH2 domain therefore blocks STAT3 activation by preventing its initial phosphorylation at Tyr705 by kinases and disrupting dimer formation (20). Currently, direct STAT3 targeting primarily focuses on inhibiting functional dimerization by blocking its SH2 domain—the pivotal mediator of STAT3 dimerization and one of the most rapidly advancing therapeutic targets in pulmonary diseases (21). The DBD of STAT3 recognizes specific target gene promoter sites with relatively high specificity. Through this nuclear DNA binding, STAT3 critically regulates cell proliferation, migration, and invasion. Inhibitors targeting the STAT3 DBD can reduce its activity by blocking this essential DNA interaction. However, developing DBD inhibitors remains challenging, including limited accessibility due to the target’s nuclear localization and the inherent difficulty in designing compounds that selectively engage its large, flat binding surface. Additionally, blocking the DBD risks disrupting transcription of genes critical for normal cellular functions, heightening potential off-target effects (9). Napabucasin (a DBD-targeted small molecule) is the only direct STAT3 inhibitor to reach phase III trials.

In its inactive state, STAT3 localizes to the cytoplasm. Upon stimulation, tyrosine phosphorylation triggers dimerization, nuclear translocation, and transcriptional activation of target genes (22). Beyond phosphorylation, other post-translational modifications on STAT3, such as acetylation, methylation, and sumoylation, have been identified. STAT3 can be acetylated at several lysine residues within both the NH2 and SH2 domains by histone acetyltransferases CBP/p300 (23). This modification enhances STAT3 activity by increasing dimer stabilization and tyrosine phosphorylation. Conversely, deacetylation mediated by histone deacetylases or sirtuin 1 can attenuate STAT3 transcriptional activity and promote its nuclear export (24, 25). Similarly, STAT3 methylation is also dynamically regulated by methyltransferases and demethylases. Lysine 49 (K49) monomethylation enhances STAT3 transcriptional activity, whereas Lysine 140 (K140) trimethylation suppresses DNA binding (26, 27). Therapeutic targeting of this methylation-demethylation balance (e.g., SET9 inhibitor) shows promise in oncological and inflammatory diseases. SUMOylation is a process in which SUMO modifies target proteins by forming isopeptide bonds with specific lysine residues. Small ubiquitin-like modifier 2/3 (SUMO2/3)-mediated sumoylation at STAT3 lysine 451 enhances its interaction with nuclear phosphatase TC45, sequestering phosphorylated STAT3 in the nucleus (28). Conversely, SENP3-catalyzed desumoylation promotes increased phosphorylation of STAT3.

**Figure 2:**
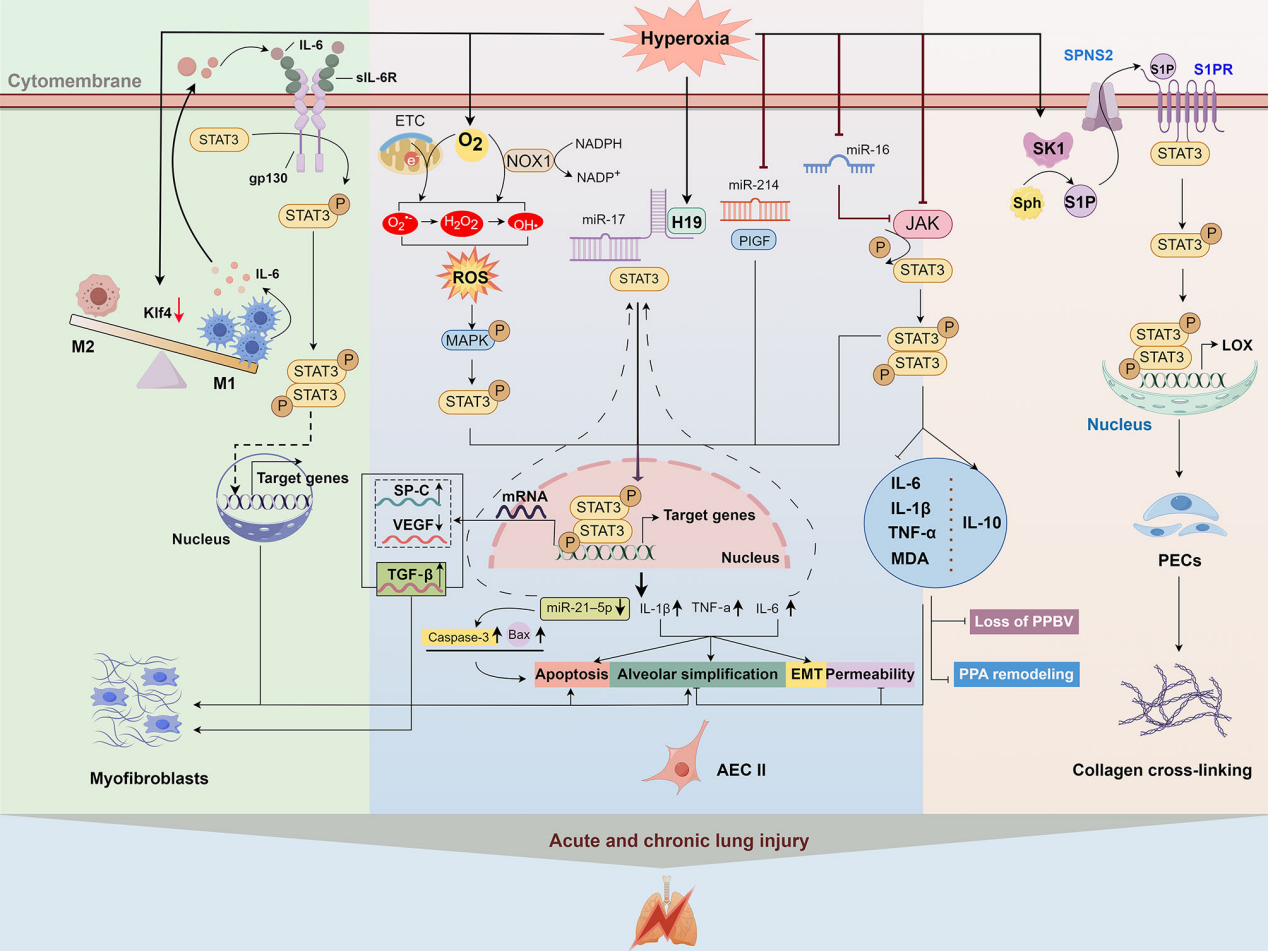
The facilitatory role of STAT3 in HLI. Hyperoxia promotes STAT3 activation through both a direct ROS-independent MAPK activation-mediated pathway and an indirect miRNA-mediated pathway. Phosphorylated STAT3 dimerizes and activates downstream target genes such as inflammatory factors (IL-6, IL-1β, and TNF-α) and miR-21, thereby promoting alveolar apoptosis, simplification, and EMT. In addition to attenuating inflammation, another non-coding RNA, lncRNA H19, is also associated with changes in different fibrotic biomarkers (VEGF, TGF-β, and SP-C) in lung tissues of BPD newborn mice via the miR-17/STAT3 axis. Hyperoxia also directly activates myofibroblasts via the macrophage-related IL-6/STAT3 axis, thereby promoting elastic fiber formation in BPD mice. This signaling pathway is also related to apoptosis and simplification of AECII cells. In addition to AECII cells and myofibroblasts, hyperoxia activates SPHK1/S1P signaling in BPD, promoting LOX expression through STAT3 phosphorylation in lung endothelial cells and increasing collagen deposition. AECII, alveolar type II epithelial cell; EMT, epithelial-to-mesenchymal transition; ETC, electron transport chain; gp130, glycoprotein 130; IL, interleukin; M, macrophage; JAK, Janus kinase; LOX, lysyl oxidase; MAPK, mitogen-activated protein kinase; MDA, malondialdehyde; NOX1, nicotinamide adenine dinucleotide phosphate oxidase 1; PECs, pulmonary endothelial cells; PPA, peripheral pulmonary arterial; PPBV, peripheral pulmonary blood vessels; ROS, reactive oxygen species; SK1, sphingosine kinase 1; S1P, sphingosne 1 phosphate; SP-C, surfactant protein C; Sph, sphingosine; SPNS2, S1P transporter; STAT3, signal transducer and activator of transcription 3; TGF-β, transforming growth factor-β; TNF, tumor necrosis factor; VEGF, vascular endothelial growth factor receptor.

STAT3 can be modulated by various factors such as kinases, cytokines, and non-coding RNAs (ncRNAs). This review focuses exclusively on HLI-associated regulators, including Janus kinase (JAK), mitogen-activated protein kinases (MAPK), sphingosine-1-phosphate (S1P), placental growth factor (PlGF), interleukin (IL)-6, and heme oxygenase-1 (HO-1) (16, 18, 29, 30). Their HLI-specific roles will be detailed in the following section. HLI-induced STAT3 expression and activation can also be regulated by various ncRNAs. These include microRNAs (miRNAs) such as miR-17, miR-214, and miR-16, as well as the long non-coding RNA (lncRNA) H19. These molecules directly target STAT3 and its signaling components, such as IL-6, PlGF, and JAK (**Figure 2**). The complexity of the STAT3 regulatory network and its dual role in HLI highlight the necessity to elucidate underlying mechanisms. Gaining insights into these mechanisms may facilitate the clinical translation of selective STAT3-targeted therapies for HLI, considering its context-dependent role in different cell types and exposure time.

## 3 STAT3 activation promotes HLI

Accumulating evidence suggests that STAT3 activation is a critical mediator of various types of acute and chronic lung injury, such as sepsis-induced acute lung injury, ischemia-reperfusion injury (IRI), BPD, and fibrosis, by promoting apoptosis, oxidative stress, and inflammation (29, 31, 32). The role of STAT3 in HLI has been extensively studied. Among them, eight publications reported the involvement of STAT3 in promoting HLI. **Table 1** summarizes the key findings on STAT3’s role in HLI. Mice, rats, AECs, and PECs were used to study HLI, with a focus on epithelial injury, fibrosis, and abnormal lung development. Oxygen concentrations ranged from 70% to 100% in mice and 60% to 95% in cell models, with exposure times of 48h to 14 days and 6h to 72h, respectively. Targeting STAT3 directly, RNA interference, and small-molecule inhibitors modulating upstream regulators are primary therapeutic strategies. **Figure 2** illustrates the signaling pathways and molecular mechanisms involved in STAT3-mediated HLI. Key components such as IL-6, the JAK/STAT3 and MAPK pathways, sphingosine-1-phosphate (S1P), and specific miRNAs play crucial roles in regulating this process.

**Table 1:** STAT3’s roles in HLI and potential therapeutic strategies.

Role in HLI	Study/ref.	Application model	Treatments	Effects of hyperoxia exposure	Mechanisms	Therapeutic strategy	Therapeutic efficacy
Facilitatory	Qin et al., 2023 (18)	Rat alveolar epithelial cells (AECs)	500 μM H_2_O_2_ for 36h	Induces apoptosis and inhibits cell growth in AECs	ROS-driven MAPK activation suppresses miR-21-5p by promoting STAT3 phosphorylation	MAPK inhibitors: pamapimod or SB239063; N-acetyl-L-cysteine	Decreases the levels of STAT3 and p-STAT3 proteins; reduces AECs apoptosis
	Mei et al., 2022 (88)	C57BL/6J mice	90% O_2_ for 48h	Exacerbates lung injury and decreases the 7-day cumulative survival rate	Hyperoxia increases TNF-α, IL-6, IL-1β, and MDA; decreases SOD and miR-12 expression	STAT3 inhibitor: S3I-201	Suppresses inflammation, regulates oxidative stress, improves lung permeability, and alleviates HLI
	Zhang et al., 2022 (81)	Newborn C57BL/6J mice	70% O_2_ for 14 days after birth	Promotes BPD progression	Upregulated lncRNA H19 competitively binds to miR-17 to elevate STAT3 and p-STAT3 levels	H19 knockdown	Reduces pro-inflammatory and profibrotic mediators and improves BPD
	Ha et al., 2022 (16)	WT and Sphk1^−/−^ neonatal mouse pups; HLMVECs	95% O_2_: 7 days (mice); 6h (HLMVECs)	Induces neonatal BPD	Hyperoxia activates SPHK1/S1P signaling, promoting LOX expression via STAT3 phosphorylation in lung endothelium	Sphk1 knockout; SPHK1 inhibitor: PF543; STAT3 knockdown	Reduces excess collagen deposition and improves alveolar development
	Hirani et al., 2022 (17)	WT and IL6^−/−^ newborn C57BL6 mice; MLE12; hAECs; macrophages	80% O_2_ for 14 days (mice); 85% O_2_ for 48h (macrophages); 85% O_2_ for 24h (MLE12, hAECs)	Impairs AECII homeostasis, disrupts elastic fiber formation, inhibits lung growth, and induces neonatal BPD	Hyperoxia-induced M1 polarization drives IL-6/STAT3-mediated AECII apoptosis and alveolar simplification	IL-6 mAb; sgp130Fc	Promotes AECII survival and lung growth
	Zhang et al., 2021 (29)	SD neonatal rats; AECs	95% O_2_ for 1 week with 24h normoxia intervals (rats); 85% for 24h (AECs)	Induces neonatal BPD	Hyperoxia induces alveolarization, AEC apoptosis, and fibrosis via the miR-214/PlGF/STAT3 pathway	miR-214 overexpression	Facilitates alveolarization
	Li et al., 2018 (77)	Primary human AECII cells	60% O_2_ for 24h	Induces cell apoptosis in AECII cells	Hyperoxia downregulates miR-16 and subsequently activates the JAK/STAT3 pathway	miR-16 overexpression	Inhibits apoptosis
	Carnesecchi et al., 2014 (43)	C57BL/6J mice; MLE12	100% O_2_ for 72h (mice); 95% O_2_ for 72h (MLE12)	Induces alveolar cell death	Hyperoxia induces STAT3 phosphorylation in a NOX1-dependent manner	NOX1 inhibitor: GKT136901, NOX1 silencing; STAT3 inhibitor: WP1066	Inhibits cell death
Inhibitory	Zhang et al., 2024 (19)	Newborn C57BL/6 mice; MLE12	85% O_2_ for 14 days; 95% O_2_ for 24h (MLE12)	Induces alveolar simplification and lung inflammation	Upregulates IL-1β, IL-6, TNF-α while downregulating SP-C, AQP5, and VEGFR2	Intranasal administration of L. reuteri and 3-IAld	Alleviates BPD by activating the IL-22/STAT3 signaling via IL-22 production
	Dong et al., 2022 (130)	SD neonatal rats	80% O_2_ for 21 days	Disrupts alveolar and pulmonary vascular development and decreases the survival rate	Inhibits the JAK2/STAT3 signaling pathway	hUC-MSCs (i.t. or i.p.)	Reduces inflammation and oxidative stress partially through JAK2/STAT3 activation; improves lung development
	Mizushina et al., 2015 (105)	NLRP3^−/−^, and IL-1β^−/−^ mice; primary AECs, macrophages, and neutrophils; MLE12 and MH-S	90% O_2_ for 72h (mice); 90% for 12h (primary AECs and macrophages); 90% for 24h (MLE12, MH-S, and neutrophils); 90% for 8h (MLE12 co-cultured with MH-S or neutrophils)	Exacerbates acute HLI and lethality in NLRP3−/− mice	NLRP3 regulates Stat3 activation by affecting inflammatory cell infiltration independent of IL-1	NA	NA
	Ao et al., 2011 (121)	MLE12	95% O_2_ for 24h	Induces cell injury and death	STAT3 inhibition causes lower ΔΨ and reduced proliferation	Vasoactive intestinal peptide	Reduces hyperoxia-induced cell growth inhibition and death
	Kolliputi et al., 2009 (113)	Lung-specific IL-6 transgenic mice; SAECs	100% O_2_ for 72h (mice); 80 μM H_2_O_2_ for 1h (SAECs)	Induces apoptosis and HALI	Hyperoxia-induced ROS triggers TNFR-1, activating ASK-1/JNK-mediated apoptosis	IL-6 overexpression	Activates IL-6R/JAK/STAT3 pathway, stimulating SOCS-1 and degrading ASK-1 to reduce apoptosis
	Zhang et al., 2006 (30)	Murine lung endothelial cells from WT, HO-1^−/−^, and STAT3^E−/−^ mice	100% O_2_ for 72h	The absence of endothelial HO-1 and STAT3 enhances hyperoxia-induced cell injury and death	STAT3 inhibition reduces HO-1, p-Akt, Bcl-2, and Bcl-xL upregulation while increasing cleaved caspase 3 expression	Overexpression of STAT3 and HO-1	Protects against hyperoxia-induced endothelial cell injury and death
	Lian et al., 2005 (123)	WT and doxycycline-treated CCSP-rtTA/(teto)_7_-CMV-Stat3C double-transgenic mice	95% O_2_ for 4.5 days	Induces inflammation and injury in the lung	Hyperoxia enhances alveolar MMP-9 and MMP-12 activity in alveoli either by inducing MMP expression or by inhibiting TIMP expression	Stat3C overexpression	Delays acute capillary leakage and neutrophil infiltration into the alveoli, and reduces mortality
	Yang et al., 2004 (110)	WT and doxycycline-treated CCSP-rtTA/(Teto)_7_-CMV-dnStat3 double transgenic mice	95% O_2_ until the terminal stage of life	Alveolar dnStat3 accelerates hyperoxia-induced cell death via Stat3 suppression	Inhibition of endogenous Stat3 activity disrupts the synergistic effect of Stat3-RARα and attenuates SP-B gene expression in AECII	NA	NA
	Hokuto et al., 2004 (117)	Non-deleted littermate control and transgenic Stat3 double knockout mice	95% O_2_ for 65h	The deletion of Stat3 leads to abnormalities in lung structure, permeability, and mechanics	Epithelial and vascular damage due to surfactant dysfunction, especially SP-B loss	Intratracheal treatment with exogenous SP-B	Improves survival and lung histology in Stat3-deleted mice

AECII, alveolar type II epithelial cell; AQP5; aquaporin 5; ASK, apoptosis signal-regulating kinase; BPD, bronchopulmonary dysplasia; ΔΨ, mitochondrial membrane potential; dnStat3, a dominant negative Stat3; HALI, hyperoxic acute lung injury; HLI, hyperoxic lung injury; HLMVECs, human lung microvascular endothelial cells; HO-1, heme oxygenase-1; hUC-MSCs, human umbilical cord-derived mesenchymal stem cells; IL, interleukin; JNK, Jun N-terminal kinase; lncRNA, long noncoding RNA; JAK, Janus kinase; LOX, lysyl oxidase; MAPK, mitogen-activated protein kinase; MH-S, murine alveolar macrophages; miR, microRNA; MLE12, murine lung epithelial cells; MMP, matrix metalloproteinase; NLRP3, NOD-like receptor family pyrin domain-containing 3; NOX1, nicotinamide adenine dinucleotide phosphate oxidase 1; PlGF, placental growth factor; RAR, retinoic acid receptor; ROS, reactive oxygen species; sgp130Fc, soluble glycoprotein 130 Fc fusion protein; SAECs, small airway epithelial cells; S1P, sphingosne 1 phosphate; STAT3, signal transducer and activator of transcription 3; STAT3E−/−, endothelial cell-specific STAT3−/− mice; SOCS-1, suppressor of cytokine signaling-1; SOD, superoxide dismutase; SP-B/C, surfactant protein B/C; SPHK1, sphingosine kinase 1; TIMP, tissue inhibitor of metalloproteinases; TNF, tumor necrosis factor; TNFR, TNF receptor; VEGFR2, vascular endothelial growth factor receptor 2.

### 3.1 JAK-STAT3 signaling pathway

The activation of STAT3 primarily relies on the activity of JAKs. The JAKs are a family of four intracellular nonreceptor tyrosine kinases (JAK1, JAK2, JAK3, and tyrosine kinase 2) that primarily transduce signals from cytokine- and growth factor-activated cell-surface receptors (33). The JAK-STAT3 pathway mediates survival, inflammation, and immune responses, and contributes to disease development in multiple organs, including the lung (34–36).

#### 3.1.1 ROS-mediated JAK/STAT3 activation

One of the key mechanisms of HLI is the generation of reactive oxygen species (ROS). Hyperoxia promotes ROS production (e.g., O_2_•− and H_2_O_2_) through high oxygen partial pressure and by causing electron leakage via inhibition of complex I and II of the electron transport chain (37) (**Figure 2**). Another major source of hyperoxia-induced ROS is the NADPH oxidase, particularly the nicotinamide adenine dinucleotide phosphate oxidase 1 (NOX1) (**Figure 2**), which is predominantly expressed in alveolar epithelial and endothelial cells (38, 39). JAK-STAT3 signaling can be activated by ROS and has been implicated in various disease development, such as oxygen-induced retinopathy, intestinal epithelial cell apoptosis, and cardiac fibrosis (40–42). Carnesecchi et al. demonstrated that NOX1-derived ROS contributes to hyperoxia-induced alveolar cell death and ARDS in mice through STAT3 activation (43). In an H_2_O_2_-simulated ROS environment, the JAK-STAT3 signaling pathway was involved in the oxidative stress-induced inhibition of the surfactant protein B (SP-B) gene (44). In addition to supplemental O_2_, tissue hyperoxia is implicated in various pathologies, such as IRI, which involves sudden bursts of O_2_ during reperfusion (45). Yu et al. showed that this process increases ROS levels and induces ferroptosis in lung IRI through the JAK2/STAT3 signaling pathway (32). Therefore, lowering ROS levels to inhibit the JAK2/STAT3 pathway may offer a therapeutic strategy for HLI.

#### 3.1.2 IL-6/JAK/STAT3 signaling pathway

Inflammation is another central mechanism in HLI. In the lipopolysaccharide (LPS)/sepsis-induced ALI model, activation of JAK-STAT3 signaling in lung epithelial cells and alveolar macrophages upregulated pro-inflammatory cytokines [IL-6, IL-1β, tumor necrosis factor-alpha (TNF-α)] and chemokines (CC chemokine receptor 2, C-X-C motif chemokine ligand 15) (46–52). This leads to increased alveolar-capillary permeability and severe respiratory failure. In addition to acute injury, JAK2-STAT3 activation also contributed to fibrosis in chronic inflammatory lung diseases (e.g., idiopathic pulmonary fibrosis and interstitial lung disease) by promoting proliferation, senescence, autophagy, endoplasmic reticulum stress, and epithelial/fibroblast to mesenchymal transition (53). These studies highlight the potential for targeting the JAK/STAT3 axis in therapeutic strategies for HLI.

Among these inflammatory cytokines, IL-6 is a central mediator of cellular communication and a key regulator of inflammatory responses. It possesses both anti-inflammatory and pro-inflammatory properties (54) and functions as both a downstream target and an upstream regulator of the STAT3 pathway. The dual role of IL-6 in inflammation is primarily mediated through classic and trans-signaling pathways, both of which require the signal-transducing receptor subunit gp130 and involve the JAK/STAT3 pathway as the primary downstream signaling (55). In classic signaling, IL-6 binds to the membrane-bound IL-6 receptor (IL-6R) and interacts with gp130 to activate JAK/STAT3 signaling (56, 57). In trans-signaling, IL-6 binds to soluble IL-6R (sIL-6R), which is generated by alternative splicing or proteolytic cleavage of IL-6R, forming IL-6-sIL-6R complex (58). Then it interacts with gp130 on cells lacking IL-6R, activating downstream pathways such as STAT3, and usually exerts a pro-inflammatory effect. This difference in IL-6-initiated pro-inflammatory and anti-inflammatory responses may relate to the expression ratio of IL-6R and gp130 in different cell types and environments (54). When gp130 exceeds IL-6R, trans-signaling dominates to exert pro-inflammatory effects. This pathway is primarily associated with pro-inflammatory responses and has been implicated in various inflammatory lung diseases, including COVID-19-related acute ARDS, pulmonary fibrosis, and asthma (59–62).

Earlier studies demonstrated that hyperoxia exposure increases IL-6 levels in the lungs of mice (63–67). IL-6 is primarily secreted by macrophages (68). Hirani et al. demonstrated that M1-like macrophage activation is linked to IL-6/STAT3 axis in clinical and experimental BPD. Inhibition of macrophage-related IL-6 trans-signaling with soluble glycoprotein 130 Fc fusion protein (sgp130Fc), an innovative therapeutic biomacromolecular drug that specifically targets IL-6 trans-signaling, enhances AECII survival and promotes lung growth in experimental BPD (17) (**Figure 2**). Therefore, targeted blockade of this pathway may offer therapeutic advantages in hyperoxia-induced BPD.

### 3.2 miRNA-mediated regulation of STAT3 signaling

miRNAs are small ncRNA molecules (18–25 nucleotides in length) that primarily regulate gene expression post-transcriptionally by binding to the 3’ untranslated region of target mRNAs, thereby inhibiting translation or promoting mRNA degradation (69). Conversely, miRNA downregulation is frequently associated with target mRNA upregulation. These molecules play critical roles in diverse biological processes, including cell differentiation, proliferation, and apoptosis. In addition, miRNAs can be regulated by lncRNAs through competing binding to target mRNAs (70). This mechanism relieves the inhibition of miRNA on target genes, thereby enhancing their expression.

#### 3.2.1 Core evidence of miRNAs targeting STAT3

Multiple miRNAs have been implicated in HLI pathogenesis in both *in vivo* and *in vitro* studies (71–76). One of the proposed mechanisms is miRNA-mediated upregulation of STAT3 signaling. In an *in vitro* HALI model, hyperoxia increased the expression of JAK and STAT3 in AECII cells, and miR-16 transfection reversed this effect (77) (**Figure 2**), indicating a potential association between miR-16 downregulation and JAK/STAT3 pathway activation in hyperoxia-induced AECII apoptosis. However, the direct target of miR-16 in the JAK/STAT3 pathway is unknown due to missing evidence for miRNA-target gene interaction. miR-214 is widely involved in the pathogenesis of various human disorders, including lung diseases (78–80). It has been shown to downregulate the expression of STAT3 in human cervical and colorectal cancer cells. In a BPD rat model, hyperoxia downregulated miR-214 expression concomitant with elevated PlGF and STAT3 activation; miR-214 overexpression attenuated this phenotype, suggesting a regulatory role in the PlGF/STAT3 axis (29). This cascade was associated with upregulated proinflammatory cytokines (IL-1β, TNF-ɑ, and IL-6) while promoting PECs apoptosis and impairing alveolarization (**Figure 2**). In neonatal mice with BPD induced by hyperoxia, STAT3/p-STAT3 upregulation can also be facilitated by H19 upregulation, which reduces the inhibitory effects of miR-17 on STAT3 by competitively binding to miR-17. Inhibition of H19 with si-H19 intervention improved this pulmonary injury via the miR-17/STAT3 axis. It relieved p-STAT3-induced inflammatory response (indicated by decreased IL-6 and IL-1β levels) and modulated fibrotic biomarkers (characterized by upregulated vascular endothelial growth factor and downregulated transforming growth factor β1) (81) (**Figure 2**).

#### 3.2.2 The miRNA–STAT3 feedback loop in HALI progression

miRNA not only regulates STAT3 but is also its target gene. Negative regulation of STAT3 by miR-21 has been reported in multiple diseases, including LPS-induced ALI (82–85). Using the dual-luciferase reporter gene experiment and miR-21 overexpression, Zhou et al. confirmed that miR-21 targeted the regulation of STAT3 and inhibited inflammatory response and apoptosis, and maintained redox balance to alleviate HALI (86). Previous studies demonstrated that hyperoxia reduced miR-21 levels, whereas miR-21 overexpression effectively attenuated HALI (87). Qin et al. showed that ROS-driven MAPK activation reduces miR-21 expression in AECII cells by activating STAT3. Conversely, inhibiting STAT3 effectively reduces apoptosis of AECII cells and ameliorates lung permeability by suppressing inflammation and oxidative stress through the upregulation of miR-21 expression (88). Therefore, the bidirectional positive feedback between down-regulated miR-21 and up-regulated STAT3 may accelerate HALI progression (**Figure 2**), and disruption of this loop may be a therapeutic target.

#### 3.2.3 Translation of miRNA therapeutics: opportunities and challenges

Circulating miRNAs have been established as potential diagnostic biomarkers in several diseases (89–91). Therefore, these four STAT3-associated miRNAs—miR-16, miR-17, miR-214, and miR-21—may serve as potential diagnostic/prognostic biomarkers for early recognition and treatment of HLI. Circular RNAs represent another type of ncRNAs. Circular RNA 406961 has been shown to regulate PM2.5-induced inflammatory responses in human bronchial epithelial cells through ILF2 interaction and subsequent STAT3/JNK pathway activation (92). Whether additional ncRNAs, including other circular RNAs, contribute to this process requires further investigation.

miRNA-based therapeutics are still in early-stage development, and currently, no miRNA-based therapeutics have received clinical approval; however, their therapeutic potential is beginning to emerge. Several therapeutics targeting miRNAs have entered clinical trials. Obefazimod, a miR-124 up-regulator, is being evaluated for moderate-to-severe ulcerative colitis (93); CDR132L, a miR-132 inhibitor, for heart failure (94); and Remlarsen, a miR-29 mimic, for cutaneous fibrosis (95). Despite their therapeutic promise, miRNA therapeutics face several challenges. First, each miRNA can regulate multiple genes, and a single gene can be co-regulated by several miRNAs; this combinatorial network complicates accurate prediction of efficacy and off-target effects (e.g., miR-21 protects against HLI yet promotes tumors) (96). Second, miRNAs are rapidly degraded by nucleases, and their hydrophilicity and negative charge make it difficult for them to penetrate the cell membrane. Additionally, achieving efficient intracellular delivery remains a challenge. Commonly used delivery methods, such as lipid nanoparticles, can trigger immune responses and often lack sufficient tissue-targeting specificity.

Recently, the miRNA-based proteolysis-targeting chimeras (PROTACs) strategy has emerged as a potential solution for achieving consistently high levels of therapeutic endogenous miRNAs (97). New nucleic acid delivery technologies with organ-specific targeting methods have been developed, facilitating the systematic application of miRNA-based PROTACs and reducing off-target effects. Combining miRNA therapy with a STAT3 inhibitor or STAT3-pathway modulators may enhance HLI treatment efficacy and minimize off-target effects by more effectively blocking the miRNA-STAT3 axis and lowering the effective dose of either therapy alone.

### 3.3 S1P-STAT3-LOX signaling axis

S1P, a bioactive lysophospholipid synthesized by sphingosine kinase 1 (SPHK1), plays a crucial role in various biological processes and diseases, including both acute and chronic lung diseases (98). Research has primarily focused on its role in pulmonary endothelial dysfunction and fibrotic processes, among which inflammation, oxidative stress, and fibroblast activation are amongst the most important mechanisms (99–103). S1P plays pleiotropic signaling roles by activating five different receptors (S1P1-5). Harijith et al. first reported the involvement of the S1P-S1P receptor 1/2 signaling axis in hyperoxia-induced lung endothelial injury via ROS production in neonatal and adult mice (104). Ha et al. later demonstrated that hyperoxia activates SPHK1/S1P signaling, promoting lysyl oxidase (LOX) expression via STAT3 phosphorylation in PECs (16). This process resulted in increased collagen cross-linking. Inhibiting this pathway and its components reduced LOX production and collagen staining in lung tissue while restoring lung alveolarization. Therefore, targeting LOX through the SPHK1/S1P/STAT3 signaling pathway may provide a novel therapeutic strategy for treating hyperoxia-induced BPD.

## 4 STAT3 activation provides protection against hyperoxia-induced lung injury

**Figure 3:**
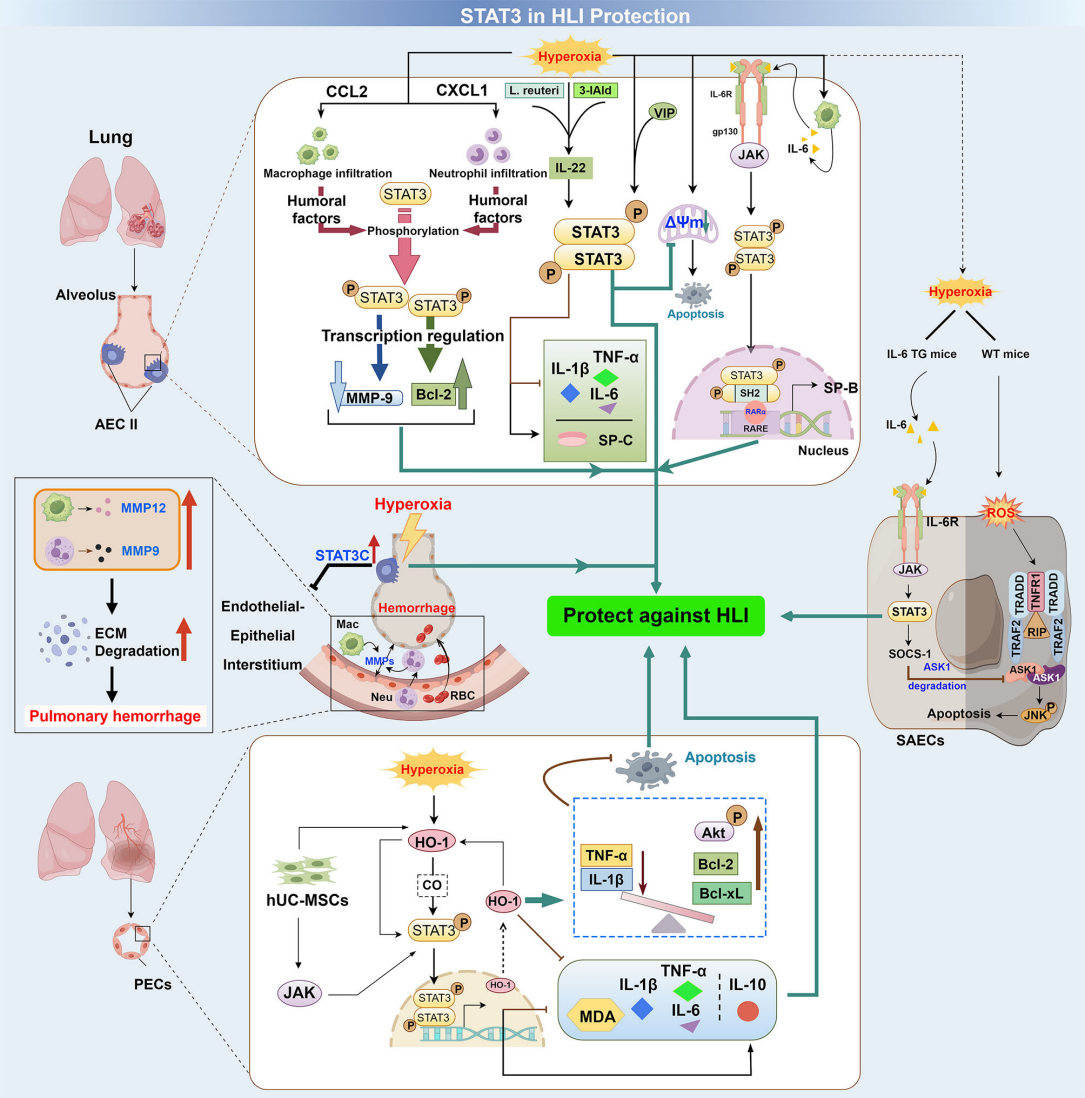
The protective role of STAT3 in HLI. In AECII cells, hyperoxia induces lung macrophage/neutrophil infiltration via CCL2/CXCL1 chemokines. This process activates STAT3, which increases Bcl-2, decreases MMP-9, and reduces alveolar apoptosis and permeability. Hyperoxia also induces macrophage IL-6 release, thereby activating the JAK/STAT3 axis. Subsequently, STAT3 upregulates SP-B gene expression via interaction with RA receptor, thereby protecting surfactant protein balance against HLI. Furthermore, the activation of the IL-22/STAT3 signaling pathway by Lactobacillus and its metabolite tryptophan alleviates hyperoxia-induced injury in AECII cells in neonatal mice. Similarly, VIP treatment prevents hyperoxia-induced decreases in ΔΨ and apoptosis in AECII cells by promoting STAT3 activation. In SAECs, exposure to high concentrations of oxygen activates the classical IL-6 receptor/JAK/STAT3 pathway, which stimulates the expression of SOCS-1 and leads to the degradation of ASK-1, ultimately reducing apoptosis. In PECs, hyperoxia upregulates HO-1 expression, which in turn activates STAT3 through both CO-dependent and -independent pathways. Subsequently, STAT3 activates HO-1, forming a positive feedback loop. HO-1 activation reduces apoptosis in PECs by increasing antiapoptotic proteins (p-Akt, Bcl-2, Bcl-xL) and decreasing pro-inflammatory mediators (TNF-α, IL-1β) as well as the apoptosis marker cleaved caspase-3. JAK/STAT3 signaling is also involved in hUC-MSCs-mediated mitigation of hyperoxia-induced PECs injury by reducing TNF-α, IL-1β, IL-6, and MDA while increasing IL-10. STAT3C reduces hyperoxia-induced capillary leakage and neutrophil infiltration, alleviating endothelial-epithelial interstitial damage and pulmonary hemorrhage by inhibiting the synthesis and release of MMP-9 and MMP-12. ASK, apoptosis signal-regulating kinase; Bcl-2, 3-IAld, indole-3-aldehyde; ΔΨ, mitochondrial membrane potential; CO, carbon monoxide; HO-1, heme oxygenase-1; hUC-MSCs, human umbilical cord-derived mesenchymal stem cells; JNK, c-Jun N-terminal kinase; MDA, malondialdehyde; MMP, matrix metalloproteinase; RA, retinoic acid; RAR, retinoic acid receptor; RIP, receptor-interacting protein; SAECs, small airway epithelial cells; SH2, Src homology 2 domain; SOCS-1, suppressor of cytokine signaling-1; SP, surfactant protein; TG, transgenic; WT, wild type; TNFR, TNF receptor; TRADD, TNFR-associated death domain protein; TRAF2, TNF receptor-associated factor 2.

In addition to its established role in promoting HLI, STAT3 has been shown to protect AECII cells and PECs from HLI (**Table 1**). The protective role of STAT3 in HLI was investigated using multiple models, including mouse lung epithelial (MLE12) cells, primary AECs, murine lung endothelial cells, newborn rodents, and genetically modified mice (e.g., CCSP-rtTA transgenic mice and endothelial STAT3-deficient mice). As shown in **Figure 3**, STAT3 is activated through hyperoxia-induced inflammatory cytokines (such as IL-6 and IL-22), chemokine-induced macrophage and neutrophil infiltration, and subsequent humoral factors production. JAK and HO-1 signaling pathways are also implicated in STAT3 activation during hyperoxia. Activating STAT3 or its upstream regulators (genetically or pharmacologically) enhanced alveolarization and vascular integrity, and boosted proliferation of AECII and PECs by mitigating inflammation and apoptosis, stabilizing mitochondrial membrane potential (ΔΨ), and suppressing hyperoxia-induced MMP-9 and MMP-12 production/release from neutrophils and alveolar resident cells. Conversely, STAT3 deficiency will exacerbate HLI. In the following sections, we will summarize the protective effects of STAT3 in HLI, focusing specifically on its roles in lung epithelial cells, PECs, and the endothelial-epithelial interstitium.

### 4.1 Epithelial cells

Mizushina et al. demonstrated that hyperoxia induces macrophage and neutrophil infiltration into the lungs via the C-C motif chemokine ligand 2 and C-X-C motif chemokine ligand 1 chemokines (105). These infiltrated inflammatory cells then promote STAT3 activation in AECII cells. This activation of STAT3 subsequently increases the transcription of Bcl-2 (an anti-apoptotic protein) while decreasing the transcription of MMP-9, a matrix-degrading proteinase linked to alveolar destruction. These changes lead to a reduction in alveolar cell apoptosis and permeability. The authors hypothesized that the STAT3 activation may be associated with macrophage- and neutrophil-derived humoral factors, as indicated by increased mRNA levels of IL-6 and leukemia inhibitory factor (Lif), both of which are recognized as cytokines that activate STAT3 (106, 107). Unfortunately, this study does not directly verify the relationship between IL-6, Lif, and STAT3 under hyperoxic conditions.

Classic IL-6R-mediated signaling is considered to have anti-inflammatory effects. Epithelial cells represent one of the limited cell populations expressing the IL-6R (108). Previous studies have indicated that the epithelial IL-6/IL-6R axis, particularly in bronchial and alveolar epithelium, exerts a protective effect against HLI by inhibiting inflammation and oxidative stress, maintaining surfactant protein homeostasis, and reducing mitochondrial damage (109–115). Among the studies, one study highlighted the role of the IL-6/IL-6R-activated JAK-STAT3 pathway in protecting against hyperoxia-induced AECII cell injury by regulating SP-B homeostasis (110). SP-B, a critical component of pulmonary surfactant, plays an essential role in epithelial cell remodeling following oxygen injury (116). An earlier study demonstrated that intratracheal SP-B improves lung histology and increases survival in STAT3-deleted mice during hyperoxia (117). It was later determined that the classical IL-6R/JAK/STAT3 pathway is responsible for regulating the production of SP-B. Furthermore, hyperoxia may induce IL-6 release from macrophages, which then binds to IL-6R, triggering JAK-mediated STAT3 phosphorylation and nuclear translocation. Activated STAT3 subsequently upregulated the gene expression of SP-B by interacting with retinoic acid (RA) receptor through its SH2 domain and activating RA response elements, thereby maintaining surfactant protein homeostasis and protecting against HLI (110) (**Figure 3**).

In another study, Kolliputi et al. showed that IL-6 lung-specific overexpression or exogenous IL-6 treatment protects against HLAI by activating the IL-6R/JAK/STAT3 pathway. This activation promotes the degradation of apoptosis signal-regulating kinase 1 through the action of suppressor of cytokine signaling (SOCS)-1, thereby reducing hyperoxia-induced apoptosis in small airway epithelial cells (SAECs) (113) (**Figure 3**). Another member of the SOCS family, SOCS-3, has been shown to prevent lung injuries caused by hyperglycemia and LPS through the inhibition of the JAK2/STAT3 pathway (118, 119). This discrepancy highlights the complexity of the SOCSs/JAK2/STAT3 signaling pathway in different models of lung injury.

In addition to IL-6, IL-22 may also serve as a key protective factor against HLI by activating the STAT3 pathway. *In vivo* experiments, Zhang et al. demonstrated that IL-22 reduces pathological alterations and inflammation in hyperoxia-induced BPD (19). Using an AECII cell model, they revealed an association between the IL-22/STAT3 pathway and this protective effect (**Figure 3**). Although IL-22 treatment increased the levels of lung aquaporin 5, an AECI cell marker, and vascular endothelial growth factor receptor 2, which regulates angiogenesis and vascular permeability, further direct evidence from AECI cells and PECs is needed to clarify the role of the IL-22/STAT3 signaling in alveolar and vascular development in hyperoxia-exposed neonatal mice.

The loss of ΔΨ is a hallmark of apoptosis (120). Ao et al. demonstrated that hyperoxia decreases ΔΨ, causing apoptosis of AECII cells (121). This process can be prevented by vasoactive intestinal peptide (VIP) treatment, which promotes the activation of STAT3 (**Figure 3**). The authors did not provide insight regarding the cause of decreased ΔΨ. We hypothesized that the ROS may be the reason. This is based on the understanding that ROS generation is a significant response to hyperoxia exposure, and it also plays a crucial role in opening the mitochondrial permeability transition pore, which can lead to a decrease in ΔΨ (122).

### 4.2 Endothelial-epithelial interstitium

The protective role of lung epithelial STAT3 in HLI is not limited to the tissue itself. Hokuto et al. demonstrated that overexpression of STAT3C (a constitutive active form of STAT3) delays hyperoxia-induced acute capillary leakage and neutrophil infiltration into the alveolar region, alleviating endothelial-epithelial interstitial damage and pulmonary hemorrhage (123). This protective effect is achieved by preventing the degradation of the extracellular matrix through a decrease in the synthesis and release of neutrophil-derived MMP-9 and alveolar macrophage-derived MMP-12 (**Figure 3**).

### 4.3 PECs

HO-1 is a key cytoprotective, antioxidant, and anti-inflammatory molecule, mainly through the removal of prooxidative heme and the production of antioxidative biliverdin/bilirubin and carbon monoxide (CO) (124, 125). HO-1 can be induced by several injurious stimuli, including hyperoxia (126). Early studies revealed the protective effects of HO-1 and CO in hyperoxia-induced vascular endothelial injury (127–129), but the mechanisms are not fully understood. Evidence suggests that STAT3 activation may be associated with the induction of the HO-1 gene under hyperoxia, and that there may exist a positive feedback system where STAT3 activation boosts HO-1 expression and *vice versa* (30) (**Figure 3**). While experimental evidence demonstrates that HO-1 activation inhibits hyperoxia-induced endothelial apoptosis by regulating apoptotic proteins (p-Akt, Bcl-2, Bcl-xL) and suppressing pro-inflammatory mediators (TNF-α, IL-1β) alongside cleaved caspase-3 (**Figure 3**), the precise interdependence between STAT3 and HO-1 requires further validation. Although there is a significant interdependence between HO-1 and STAT3, endothelial STAT3 appears to protect endothelial cells through both HO-1-dependent and HO-1-independent mechanisms. This is supported by two findings: the protective effects of HO-1 and CO are eliminated in mice with conditional endothelial STAT3 deletion; and STAT3 overexpression can partially rescue HO-1-deficient lung endothelial cells from hyperoxia-induced apoptosis. Preliminary evidence suggests that HO-1 and JAK/STAT3 signaling might contribute to human umbilical cord-derived mesenchymal stem cells (hUC-MSCs)-mediated mitigation of hyperoxia-induced lung endothelial high permeability, loss of peripheral pulmonary blood vessels, and peripheral pulmonary arterial remodeling, as well as alveolar simplification (130). This is achieved by decreasing inflammatory and oxidative responses, characterized by lower levels of TNF-α, IL-1β, and IL-6, along with decreased malondialdehyde and increased IL-10 (**Figures 2**, **3**). Collectively, current evidence positions endothelial STAT3 as a therapeutic target for hyperoxia-induced vascular injury, pending further mechanistic validation.

## 5 Insights into the dual regulatory effects of STAT3 on HLI

First, the different roles of STAT3 may be partly attributed to variations in oxygen concentration and exposure duration. At identical oxygen concentrations, divergent STAT3 functions emerge across exposure times. For example, STAT3 activation protects MLE12 cells exposed to 95% O_2_ for 24h from injury and death (121), whereas extended exposure (72h) at this concentration shifts STAT3 signaling toward apoptosis (43). Similarly, varying oxygen concentrations over fixed exposure durations can lead to opposing STAT3-mediated outcomes. Neonatal mice exposed to 95% oxygen for 7 days experienced STAT3-driven injury (88), while a shorter exposure of 4.5 days at the same oxygen level allowed for STAT3-dependent protection (123).

Second, different developmental stages may also contribute to the distinct roles of STAT3. In adult mice, pulmonary IL-6 overexpression attenuates hyperoxic injury, reduces cell death, and improves survival (109). Conversely, elevated IL-6 increases mortality, promotes elastic fiber deposition, and impairs lung development in newborns (17, 131). The developmental differences in the responses of IL-6 may explain the distinct roles of STAT3. As detailed in section 3.1, IL-6’s dual inflammatory role may arise from varying IL-6R/gp130 expression ratios. However, this hypothesis requires evaluation during distinct developmental stages under hyperoxia.

In addition, different cell types may also yield divergent outcomes. In AECII cells, activation of the JAK/STAT3 signaling pathway promotes apoptosis induced by hyperoxia (77). In contrast, when this signaling pathway is activated in PECs and SAECs, it promotes lung development and prevents apoptosis (130). The discrepancy may be attributable to two key mechanisms. First, AECs express minimal membrane-bound IL-6R (mIL-6R) but high gp130 levels. Conversely, PECs express abundant mIL-6R, enabling classical IL-6 signaling that triggers STAT3-mediated cytoprotection. Second, during endothelial injury, hemoglobin-derived heme activates HO-1 in PECs. This enzyme eliminates pro-oxidative heme and produces antioxidative bilirubin while simultaneously initiating a STAT3-dependent protective loop in PECs.

Furthermore, activation of STAT3 by different upstream signals may lead to different biological effects. For example, when upstream signaling includes pro-inflammatory signals, such as epithelial MAPK and endothelial S1P under hyperoxic conditions, STAT3 activation appears to promote lung injury (16, 18). On the other hand, the activation of STAT3 by anti-inflammatory mediators such as IL-22 provides protective effects. Beyond MAPK and S1P signaling, STAT3 engages other pathways to mediate pro- and anti-inflammatory responses. STAT3 collaborates with the cGAS-STING pathway to promote inflammatory cell death (132). In the cytoplasm, STAT3 also influences various signaling pathways by interacting with proteins such as NF-κB and the PI3K-Akt signaling pathway, which affects both cell survival and inflammation (133, 134). Although not reported in HLI, common pathophysiological features—particularly oxidative injury and immune dysregulation—suggest these pathways could be involved. Understanding how STAT3 interacts within transcriptional networks may clarify its context-dependent functions and lead to new therapeutic strategies in HLI.

These complex and orderly regulatory mechanisms of STAT3 contribute to its dual role in HLI, forming the basis for its various therapeutic effects on HLI in different cell types and disease contexts. Furthermore, these insights facilitate developing a predictive model using development stage, exposure duration, and specific biomarkers (e.g., cytokines like IL-6R:gp130 ratio, ncRNAs such as miRNAs, and STAT3-regulated proteins) to identify patients who may benefit from STAT3-targeted therapies.

## 6 Challenges and limitations of current STAT3-targeted therapy in HLI

STAT3 inhibitors can be divided into direct (e.g., targeting STAT3 dimerization, SH2 domain, or DNA binding) or indirect (e.g., modulating upstream regulators like JAK, MAPK, and IL-6) inhibitors. Current applications of STAT3 inhibitors in cancer therapy indicate that small-molecule inhibitors often have several drawbacks, including poor selectivity and specificity, inadequate cellular permeability, low bioavailability, and off-target effects. For HLI therapy, this dilemma remains. S3I-201, an SH2 domain inhibitor, exhibits limited affinity and selectivity due to homology with other STAT proteins (135). Low bioavailability and poor aqueous solubility also restrict oral delivery. In addition, a higher dose is required to achieve therapeutic levels, which increases the risk of off-target effects. These limitations currently hinder its advancement to clinical trials. WP1066 is a potent JAK2/STAT3 inhibitor with good oral bioavailability and the ability to penetrate the blood-brain barrier (136). These characteristics have led to its use in several clinical trials aimed at treating brain tumors. Research on WP1066 in pulmonary disease primarily focused on inhibiting inflammation and suppressing abnormal proliferation, but was limited to preclinical studies (137, 138). Notably, WP1066 exerts broad effects on multiple STAT proteins and extracellular signal-regulated kinase 1/2. This extensive inhibition may lead to immune suppression and hematological toxicity, as observed with other JAK inhibitors. Similarly, the MAPK inhibitors often exhibit unstable therapeutic efficacy or off-target toxicity due to the intricate involvement of the p38 pathway in immune regulation (139). Both the SPHK1 inhibitor PF543 and the NOX1 inhibitor GKT136901 encounter challenges such as short half-life, poor water solubility, and potential off-target effects, which make their clinical applications difficult (140, 141).

Gene therapies (e.g., miRNA interference, gene knockdown or overexpression) targeting STAT3 or its upstream regulators also encounter significant challenges. A principal concern is the risk of off-target effects, as these genes participate in extensive signaling networks; their knockdown or overexpression may inadvertently dysregulate non-target pathways. Although preclinical data demonstrate short-term benefits, the long-term efficacy and safety of HLI treatment require rigorous validation in human trials. Resolving these limitations is essential for establishing STAT3-targeted gene therapy as a clinically viable option for HLI.

sgp130Fc, a selective inhibitor of IL-6 trans-signaling, is undergoing clinical evaluation for ulcerative colitis (UC) and shows therapeutic promise in lung diseases (142). It blocks pro-inflammatory signaling while preserving membrane-bound IL-6 pathways, thus avoiding systemic adverse effects linked to IL-6’s pleiotropic functions in immunity, metabolism, and inflammation. While clinical trials are underway for UC, development for other diseases, including lung diseases, remains confined to animal models. Furthermore, screening patients with elevated sIL-6R expression to enable precision therapy poses challenges. The bioactive peptides (such as VIP and SP-B) and probiotic Lactobacilli both face significant challenges in oral bioavailability and pulmonary delivery efficiency.

In addition to the limitations previously mentioned, several others should be noted as well. First, most studies have primarily focused on the role of STAT3 in hyperoxia-induced neonatal BPD. This narrow focus may limit the applicability of their findings to other populations and types of HLI. Second, the studies listed in **Table 1** are restricted to either *in vivo* or *in vitro* experiments, limiting their translational utility for clinical applications. Using advanced experimental models such as human organoid models or lung-on-a-chip, which offer a higher degree of human physiological relevance than traditional animal or cell models, could provide critical insights into the posttranslational modifications of STAT3 and its signaling interactions in human lung tissue. These insights are essential for facilitating clinical translation and thereby filling this gap. Currently, no clinical studies have correlated STAT3 with HLI. Even so, several STAT3-targeting inhibitors are under clinical evaluation for pulmonary diseases. Furthermore, STAT3 exerts both protective and detrimental effects in HLI, making it challenging to determine its exact role due to the absence of predictive biomarkers. Fourth, the regulation of HLI by STAT3 mainly concentrates on its phosphorylation, with much less focus on other posttranslational modifications like acetylation, methylation, and sumoylation, which provides a promising avenue for further exploration in HLI.

## 7 Conclusions and future directions

STAT3 is excessively activated in both acute and chronic HLI and acts as a central signaling node for PECs, myofibroblasts, and epithelial cells, especially AECII cells. Despite advancements in STAT3-targeted therapies, only a limited number of STAT3 inhibitors have advanced into clinical trials. The structural conservation of the SH2 domain enables many small-molecule STAT3 inhibitors to nonspecifically inhibit other STAT proteins, particularly STAT1 and STAT5, resulting in systemic toxicity and off-target effects. Since STAT3 signaling is regulated by molecules such as miRNAs, IL-6, HO-1, MAPK, and JAK2, small-molecule drugs that selectively target these pathways may effectively modify STAT3 activity, thereby reducing inflammation, oxidative damage, and apoptosis. Again, insufficient selectivity and specificity are the major issues of small molecules due to poor permeability, low bioavailability, and redundant signaling pathways, limiting their therapeutic potential.

Further research should be directed at improving the specificity and safety of STAT3-targeted therapies to minimize potential off-target effects. Organ-specific delivery approaches (e.g., inhalation), more efficient drug delivery systems (e.g., nanomaterial-based platforms), and novel therapeutic strategies (e.g., PROTAC-based targeted degradation) may help overcome these limitations by improving bioavailability, reducing systemic toxicity, and ensuring targeted drug delivery. Currently, U.S. Food and Drug Administration (FDA)-approved STAT3 inhibitors primarily target the dimerization of STAT3 by binding to the STAT3 SH2 domain and are restricted to cancer therapy. Therefore, exploring additional regions of STAT3 beyond its SH2 domain may help to provide a potential therapeutic target for HLI therapy. Additionally, lowering the dose of STAT3 inhibitors and combining them with antioxidant and anti-inflammatory therapies, or thoroughly validating any off-target effects, may improve therapeutic efficacy while reducing adverse effects. Moreover, due to the challenges in developing direct STAT3 inhibitors or activators and the absence of specific small-molecule options in clinical trials, repurposing existing drugs (e.g., pyrimethamine and celecoxib), natural compounds (e.g., VIP and 3-IAld), or clinically trialed agents for pulmonary conditions (e.g., silibinin or hUC-MSCs) may offer a feasible approach for HLI treatment. STAT3 exhibits a multifaceted role in mediating cell damage and protection during hyperoxia, which underpins its diverse effects on the treatment of HLI across various cell types and disease contexts. Therefore, there is an urgent need to identify predictive biomarkers to help determine which patients may respond differently to STAT3-targeted treatments. Machine learning methods may be helpful to address the issue by enhancing the processing of high-dimensional biomarker data. This approach enables more effective identification of complex patterns and integration of multi-source information (e.g., miRNAs, cytokine profiles, and clinical parameters), thereby delivering more precise individualized risk predictions. However, applications of this methodology remain underexplored.

In summary, despite the challenges and limitations, recent advances in understanding the STAT3 signaling pathway have highlighted the potential of therapeutic strategies that target STAT3 or its mediators in treating HLI. Further investigations are necessary to optimize their pharmacological properties, clinical applicability, and biomarker-driven stratification.
